# Compositional Characteristics of Macadamia Nuts and Oils from Major Growing Regions in China: A Static Evaluation

**DOI:** 10.3390/foods15152596

**Published:** 2026-07-24

**Authors:** Caifeng Yan, Ying Liu, Juan Li, Jiaojiao Yin, Xinghe Zhang, Pan Gao

**Affiliations:** 1 Key Laboratory of Edible Oil Quality and Safety, State Administration for Market Regulation, Key Laboratory for Deep Processing of Major Grain and Oil of Ministry of Education in China, College of Food Science and Engineering, Wuhan Polytechnic University, Wuhan 430023, China; 18707157452@163.com (C.Y.); liuying20219@163.com (Y.L.); yinjiaojiao@whpu.edu.cn (J.Y.); zhangxinghe_wx@163.com (X.Z.); 2 Wuhan Institute for Food and Cosmetic Control, Wuhan 430012, China; lijuanice81@163.com

**Keywords:** macadamia oil, cultivars, cultivation areas, minor constituents, antioxidant capacity

## Abstract

China’s macadamia industry has expanded remarkably; however, a comprehensive understanding of the interaction between cultivar and geographical origin in shaping oil quality remains limited. This gap constrains the development of high-value products. To address this, we conducted a systematic and comparative evaluation of macadamia nuts and their pressed oils from 15 representative cultivars across Yunnan, Guangxi, and Guangdong. Our analysis encompassed physicochemical properties, fatty acid profiles, key minor components (tocopherols, squalene, and polyphenols), and antioxidant activities. Oleic acid (56.66–62.75%) and palmitoleic acid (12.24–18.73%) were the dominant fatty acids, with Yunnan oils exhibiting the highest unsaturated fatty acid content (80.86%). Multivariate analyses revealed that geographical origin was a more dominant factor than cultivar in shaping the overall compositional profile, particularly for the minor bioactive components. A strong positive correlation was established between antioxidant capacity and the contents of polyphenols, squalene, and tocopherols. The Guangxi A4 cultivar emerged as the premier material due to its superior fatty acid profile, high bioactive content (squalene: 329.01 mg/kg; tocopherols: 64.10 mg/kg), and potent antioxidant capacity. Our findings provide a scientific foundation for the selective breeding and origin-based standardization of premium macadamia oils in China.

## 1. Introduction

Macadamia (*Macadamia integrifolia*) is commonly known as the macadamia nut or Queensland nut. It is an economically important cash crop grown in tropical and subtropical regions [1]. Since its introduction in the 20th century, China’s industry has grown significantly, establishing a major production belt in Yunnan, Guangxi and Guangdong provinces [2]. The edible kernel is prized for its delicate crisp texture and rich nutritional profile. Recognized as a premium woody oil crop, macadamia oils (MOs) are rich in unsaturated fatty acids (UFAs, >80%), predominantly oleic acid (C18:1) and palmitoleic acid (C16:1) [3]. The high monounsaturated fatty acid (MUFA) content of MOs is associated with lipid-lowering, antioxidant, and anti-inflammatory effects, benefiting gut health, immunity, and skin health, including skin renewal, scar reduction, and protection against sun damage [4,5].

MO quality is governed by the combined effects of genetic factors and the growing environment. At the molecular level, these phenotypic differences are underpinned by distinct genetic architectures of lipid biosynthesis. *M. integrifolia* possesses the highest copy number of Fata/FatB genes, which endows it with superior fatty acid synthesis capacity [6], while the chromosome-level genome of *M. tetraphylla* has further identified 33 key enzymes involved in oil biosynthesis, providing genomic clues to the species’ extraordinarily high oil content (up to 80%) [7]. However, environmental conditions are equally critical, modulating both oil quality and accumulation processes. Temperature (15–25 °C) favors uniform fruit development and lipid synthesis [8]; water availability affects ripening rate and may accelerate or delay oil synthesis under stress, thereby altering fatty acid profiles; and soil properties—optimal annual mean 19–23 °C, rainfall >1000 mm, altitude <1300 m, and soil depth >1 m, pH 4.5–6.5—collectively shape the final product [9,10]. Moreover, a significant genotype-by-environment (G × E) interaction exists: Shuai et al. demonstrated that the same cultivar exhibits compositional variations under different ecological conditions, particularly in lipid concomitants such as tocopherols and sterols, which are significantly influenced by climate and soil [11,12]. Despite this recognition, current studies often suffer from limited sample representativeness—some include cultivars from only a single province or too few cross-regional varieties—thus limiting the generalizability of findings. This knowledge gap not only impedes the formulation of region-specific production standards and informed consumer choices but also constrains a mechanistic understanding of the causes underlying regional MO variations.

This study systematically collected 15 representative macadamia cultivars from Guangxi, Guangdong, and Yunnan. We conducted a comprehensive analysis of kernel physicochemical properties and oil quality, including nutritional composition, risk factors, and oxidative stability. We hypothesized that while cultivar defines the fundamental fatty acid architecture, geographical origin would predominantly modulate the synthesis and accumulation of lipid-soluble antioxidants (e.g., tocopherols, squalene, and polyphenols) via genotype-by-environment interactions, thereby ultimately determining the oil’s oxidative stability and nutritional value. We aimed to identify superior varieties with comprehensive quality suitable for the development of high-grade edible oil. Using hierarchical cluster analysis (HCA) and principal component analysis (PCA), we elucidated the differential characteristics and population similarities among oils from different varieties and regions. The findings provide a solid data foundation for establishing a raw material selection system for MO and offer a systematic scientific basis for promoting industrial application and constructing a characteristic edible oil system.

## 2. Materials and Methods

### 2.1. Samples and Reagents

Fifteen kinds of Macadamia nuts produced in three main producing areas of Guangxi (N 20°54′~26°24′; E 104°26′~112°04′), Guangdong (N 20°13′~25°31′; E 109°39′~117°19′) and Yunnan (N 21°08′~29°15′; E 97°31′~106°11′) from September to October 2023 were collected. The Guangxi cultivation area is primarily located in hilly terrain below 1200 m in elevation, with annual rainfall exceeding 1000–2800 mm and sunshine duration over 1600 h. It features a rain-heat-synchronous climate and long frost-free periods. The Yunnan production region, situated at altitudes of 600–1500 m, receives annual precipitation of 1200–1600 mm and sunshine duration of 2000–2800 h. It is characterized by a low-latitude plateau climate with warm winters, cool summers, abundant sunlight, and generally low wind speeds [13]. Orchards in Guangdong are distributed at elevations of 500–1000 m, with annual rainfall ranging from 1500 to 2000 mm under a subtropical monsoon climate, although detailed sunshine data remain insufficient [14].

All samples were fresh and mature shelled fruits and then dried in a continuous plate dryer at 25 °C, 35 °C, and 45 °C for 12 h at each temperature stage to reduce the moisture content below 5.0%. The nuts were transported to the laboratory immediately and dehulled. Subsequently, the shelled nuts were dried at 40 °C for 12 h until achieving a moisture content below 2.0%. The samples were sealed in a polyethylene bag and stored in a cool and dry place.

The standards of 37-fatty acid methyl esters, tocopherols (*α-,β-,γ-,δ*-tocopherols, purity > 95%), 1,1-diphenyl-2-picrylhydrazyl (DPPH, purity > 97%), 2,2′-azinobis (3-ethylbenzothiazoline-6-sulfonic acid) diammonium cation (ABTS, purity > 98%), 2,4,6-tris (2-pyridyl) triazine (TPTZ, purity > 99%), (±)-6-Hydroxy-2,5,7,8-tetramethylchromane-2-carboxylic acid (97%), and gallic acid (purity > 98%) were purchased from Sigma-Aldrich (St. Louis, MO, USA). Ammonium hydroxide, potassium hydroxide, ethyl acetate, isooctane, methanol, glacial acetic acid, ether, soluble starch, chloroform and other analytical grade reagents were purchased from Sinopharm Chemical Reagent Co., Ltd. (Shanghai, China). Acetonitrile, methanol, and other analytical grade reagents were supplied by Kemiou Chemical Reagent Co., Ltd. (Tianjin, China).

### 2.2. Physiochemical Indexes of Macadamia Nut Kernel and MOs

A total of 500 g of nut kernels were crushed by a high-speed pulverizer (RHP-100, Yongkang Ronghao Industry and Trade Co., Ltd., Yongkang, China), and the crude oil was obtained by screw press (Z300, Yijiayi Machinery Equipment Co., Ltd., Anlu, China). The crude oil was centrifuged at approximately 7900× *g* for 15 min, and the upper oil phase was separated after standing to obtain the product oil. That yield was determined gravimetrically based on the following formula:

(1) $$Lipid\;yield(\%)=\frac{A_{1}}{A_{2}}\times 100\%$$

where A_1_ is the mass of MO (g) and A_2_ is the mass of Macadamia nuts (g).

The moisture content of the Macadamia nuts was determined by desiccation in a vacuum oven at 100 °C to a constant weight according to the ISO 662:2016 [15]. The fat content was determined by Soxhlet extraction, according to the AOAC method 920.39. The reducing sugar content was determined according to the GB 5009.7-2016 [16].

Acid value (AV) and peroxide value (POV) were evaluated according to the AOCS official methods Cd 3d-63 and Cd 8b-90. The extinction coefficients at 232 nm (K232) and 268 nm (K268) were determined according to the ISO 3656:2011 [17] standard method. The iodine value (IV) was determined by a calculation based on the fatty acid profile, following the AOCS official method Cd 1c-85.

### 2.3. Fatty Acid Profile

The fatty acid profile was determined according to the ISO 12966-2:2017 [18], using an Agilent 7890A gas chromatography (GC) system (7890A, Agilent, Santa Clara, CA, USA). Briefly, 0.1 g of the sample was added to 2 mL of 0.5 mol/L KOH-methanol in a 10 mL centrifuge tube and oscillated in a 60 °C water bath for 30 min. After adding 2 mL of boron trifluoride solution and vortexing for 3 min, the mixture was sequentially treated with 2 mL of saturated NaCl and 2 mL of *n*-hexane. Following 5 min of ultrasonic extraction (37 kHz, 300 W), the supernatant was filtered through a 0.22 μm organic membrane into a 1.5 mL brown vial for analysis. The injector and detector temperatures were 270 °C and 280 °C, respectively. The oven temperature program was: 100 °C for 13 min; raised at 10 °C/min to 180 °C, held for 6 min; raised at 1 °C/min to 200 °C, held for 20 min; and raised at 4 °C/min to 230 °C, held for 10.5 min. High-purity nitrogen (99.999%) was used as the carrier gas at 1 mL/min with a 100:1 split ratio. Fatty acids were identified by comparison with a 37-fatty acid methyl esters standard.

### 2.4. Minor Constituents 

#### 2.4.1. Tocopherol Content

The tocopherol content was determined according to ISO 9936:2016 [19], using a reversed-phase high-performance liquid chromatography (HPLC) system (1260, Agilent, Santa Clara, CA, USA) equipped with an ultraviolet detector. The 1.0 g oil sample was placed in a 25 mL brown centrifuge tube and dissolved in 10 mL of ammonium hydroxide to oscillate. Then, the mobile phase was diluted to the scale and continued to oscillate evenly. It was passed through a 0.22 μm organic filter membrane to a 1.5 mL brown sample bottle for testing. Separation was carried out on a Nucifera Si 60 column (4.6 mm × 250 mm, 5 μm, Hanbon, Huaian, China) at 30 °C. The mobile phase consisted of *n*-hexane and isopropanol (98.5:1.5, *v*:*v*), with a flow rate of 1 mL/min. Absorbance was measured at 295 nm, and concentrations were calculated using the standards. Results were expressed in mg/kg.

#### 2.4.2. Squalene Content

Squalene contents were determined using a GC system equipped with a HP-5 capillary column (0.25 µm, 30 m × 0.32 mm) and an FID (Agilent 7890A, Agilent Technologies, Santa Clara, CA, USA). Briefly, 0.2 g of oil was saponified with 50 mL of KOH-ethanol solution at 80 °C for 50 min. After cooling, the mixture was transferred to a separatory funnel and extracted with 150 mL of petroleum ether. The upper organic layer was collected, washed with 25 mL of 10% ethanol until neutral, and then dehydrated by passing through anhydrous sodium sulfate. The extract was concentrated to near dryness in a 40 °C water bath, reconstituted in chromatographic *n*-hexane, and diluted to 10 mL. The solution was filtered through a 0.22 μm organic membrane prior to GC analysis. Separation was performed on an HP-5 capillary column (30 m × 0.32 mm × 0.25 μm, Agilent, Santa Clara, CA, USA) with an injector temperature of 250 °C. The oven temperature program was as follows: 160 °C to 220 °C at 20 °C/min, then to 280 °C at 5 °C/min (hold 20 min), and finally to 300 °C. Helium was used as the carrier gas with a split ratio of 10:1. Detection was carried out using an FID with hydrogen and air flow rates set at 40 mL/min and 450 mL/min, respectively. The concentrations were calculated using the standards and results were expressed in mg/kg.

#### 2.4.3. Polyphenol Content

A total of 2.0 g of MO was dissolved with 6 mL of *n*-hexane in a 15 mL centrifuge tube. The solid phase extraction column (SPE, Agela Technologies, Tianjin, China) was activated with 10 mL n-hexane and 10 mL methanol in turn, and the sample flow rate was controlled at 1.0 mL/min. After the sample was loaded, it was eluted with 10 mL methanol twice, and the eluent was collected for later use. The 5 mL eluent was mixed with 0.5 mL Folin–Ciocalteu reagent, and the reaction was carried out in the dark for 3 min. Then, 1 mL 7.5% sodium carbonate solution was added, and the solution was diluted to 10 mL with methanol, and the reaction was continued in dark for 2 h. The absorbance was measured at 760 nm wavelength with distilled water as blank control. The results were expressed as mg of the gallic acid equivalent of per kg MO sample (mg/kg).

### 2.5. Free Radical Scavenging Capacity

#### 2.5.1. Oxidative Stability Index (OSI)

OSI was evaluated according to ISO 6886:2016 [20]. Using the ultrapure water ultrasonic treatment experimental tool with conductivity ≤ 0.25 μs/cm, a 3.0 g oil sample was put into the sample tube, heated at 120 °C, and the inlet air flow was controlled at a speed of 20 L/h.

#### 2.5.2. DPPH Assay

The DPPH assay analyzed the ability to reduce the stable radical through determination of the decrease in absorbance. The DPPH assay was conducted according to the method described in our previous study [21].

Polar DPPH free radical scavenging ability: a 3.0 g oil sample was mixed with 5 mL methanol in a 10 mL brown bottle. A vortex oscillator was used to fully oscillate at 2000 r/min for 15 min, and stood in the dark to collect the supernatant after stratification. The above extraction operation was repeated 3 times, and all the supernatant was combined as a polar extract for later use. The mixture was allowed to react in the dark at room temperature for 2 h, and the absorbance was then measured at 517 nm using a methanol blank as the reference.

Nonpolar and whole oil DPPH free radical scavenging ability: 0.15 g of each sample was diluted in 10 mL of ethyl acetate. Subsequently, 2.0 mL of the diluted solution was mixed with 2 mL of DPPH-ethyl acetate solution, incubated in the dark at room temperature for 2 h, and the absorbance was recorded at 517 nm against an ethyl acetate blank. A standard curve was constructed using Trolox concentration versus scavenging rate, the antioxidant activity was expressed as μmol Trolox equivalents (TE)/kg, and the scavenging rate (R) was calculated as follows:

(2) $$R=\left(1-\frac{A_{2}-A_{3}}{A_{1}}\right)\times 100\%$$

where A1 is 2 mL DPPH-methanol solution/DPPH-EA solution + 2 mL methanol/EA solution; A2 is 2 mL DPPH-methanol solution/DPPH-EA solution + 2 mL oil sample methanol extract/oil sample EA diluent solution; and A3 is 2 mL oil sample methanol extract/oil sample EA diluent + 2 mL methanol/EA solution.

#### 2.5.3. FRAP Assay

A polar extract of macadamia nut oil samples was prepared by dissolving 300 μL of the polar extract in 5 mL of methanol and mixing thoroughly to obtain the diluted solution. Then, 2 mL of the FRAP reagent and 2 mL of the diluted oil sample were added to a 10 mL centrifuge tube, followed by dilution to the mark with distilled water. After standing at room temperature in the dark for 10 min, the mixture was centrifuged at 10,000 r/min for 10 min. Using methanol as the blank, the absorbance was measured at 593 nm. The antioxidant capacity was expressed in Trolox equivalents (TE), with the unit being μmol TE/kg.

#### 2.5.4. ABTS Assay

The ABTS stock solution was diluted with methanol to an absorbance of 0.700 ± 0.020 before determination. Then, 100 μL of polar extract was mixed with 4 mL of methanol, and then 2 mL of methanol diluent of oil sample was mixed with 2 mL of ABTS working solution and placed in the dark at room temperature for 20 min for determination. The detection wavelength was 734 nm, and methanol solution was used as a blank control. The antioxidant activity was expressed as μmol Trolox equivalents (TE)/kg, and the scavenging rate (R) was calculated as follows:

(3) $$R=\left(\frac{A_{2}-A_{1}}{A_{2}}\right)\times 100\%$$

where A1 is 2 mL ABTS working solution + 2 mL oil sample methanol diluent and A2 is 2 mL ABTS working solution.

### 2.6. Hazardous Substances

The content of 3-monochloropropane-1,2-diol esters (3-MCPDE) and Aflatoxins B1 and B2 were determined by ISO 18363-4: 2021 [22] and ISO 16050:2003 [23].

Benzo(a)pyrene (BaP) content was determined following the BS EN 16619:2015 [24], with slight modifications. In brief, 0.4 g of the MO sample was added to 5 mL of n-hexane to prepare the test solution by vortex mixing. The solid phase extraction column (SPE, Biocomma, Shenzhen, China) was activated with 5 mL dichloromethane and 5 mL n-hexane in turn. Then, 10 mL *n*-hexane was used to remove impurities, and then 5 mL dichloromethane was used to elute the target. The eluent was concentrated to dryness by nitrogen blowing at 40 °C, redissolved with 1 mL acetonitrile, filtered by 0.22 μm organic filter membrane and injected for analysis. Chromatographic conditions: InertSustain-C18 reversed-phase chromatographic column (4.6 mm × 250 mm, 5 μm, Shimadzu, Beijing, China), mobile phase of acetonitrile–water (88:12, *v*/*v*), flow rate of 1.0 mL/min; the parameters of water’s 2475 fluorescence detectors were set as excitation wavelength of 384 nm and emission wavelength of 406 nm, respectively, and the injection volume was 20 μL.

### 2.7. Data Statistics and Analysis

All experiments were performed in triplicate, and data are presented as mean ± standard deviation. Data organization was carried out using Microsoft Excel (Microsoft, Redmond, WA, USA). All the experimental data were standardized using Statistical Product and Service Solutions (SPSS) 23.0 (IBM, Armonk, NY, USA). Statistical analysis was performed using Origin 2024 (OriginLab, Northampton, MA, USA) and SPSS 27 (IBM, Armonk, NY, USA). The correlations were calculated by multiple linear regression analysis using a stepwise method. The use probability of F values, P-to-enter new variables into and P-to-remove new variables from the statistical model, was *p* < 0.05 and > 0.10, respectively. In this study, HCA was applied to comprehensively evaluate physicochemical indices, nutritional components, hazardous substances, and fatty acid profiles. Data similarities and differences were systematically analyzed using dual clustering algorithms: cosine-based clustering and centroid-based clustering.

## 3. Results and Discussion

### 3.1. Physicochemical Properties of Macadamia Nut Kernels

The basic physical and chemical indexes of 15 kinds of macadamia nut kernels such as moisture, crude protein, crude fat, and reducing sugar are shown in Table 1. Moisture content significantly impacts the storage stability of macadamia nuts, potentially altering the texture, color, flavor, aroma, shelf life, and consumer acceptability of both processed and raw nuts [25]. As summarized in Table 1, the moisture content of Macadamia nuts from all producing areas and varieties is stable at the level of 1%, which meets the quality standard requirements of MOs. Significant differences in the contents of major nutritional components were observed among different macadamia varieties. Crude fat content was generally high, ranging from 65.93% (Guangxi Guire-1) to 72.38% (Guangxi A16), representing the most abundant component in the kernel. Crude protein content ranked second, exhibiting a wide range of variation, from 4.54% in Guangdong Guirui-1 to 12.97% in Guangxi A16. Reducing sugar content was the lowest overall, ranging from only 2.11% (Guangxi Guire-1) to 4.86% (Yunnan 788). The highest iodine value was found in Yunnan 791 (87.46 g /100 g) and the lowest in Guangxi OC (84.72 g /100 g), with Yunnan A4 (85.74 g/100 g) slightly lower than Guangxi A4 (86.45 g/100 g), and the greater variation in Yunnan samples indicates that genetic and climatic factors significantly affect oil unsaturation. Among the tested varieties, Guangxi A16 demonstrated the most outstanding nutritional quality, with the highest levels of both crude fat and crude protein, followed by Guangxi A4, which reached 71.15% and 11.79%, respectively. In terms of regional comparison, the average crude protein content of macadamia nuts from Guangxi (11.12%) was significantly higher than that from Guangdong (8.80%) and Yunnan (4.60%). This superiority may be attributed to the warm, humid, and sunlit climate of Guangxi, which extends the plant-growing period and promotes protein synthesis [26]. Macadamia nuts from Guangdong ranked first in average reducing sugar content (3.81%). This is likely because perennial high temperature and humid climatic conditions in Guangdong may accelerate the photosynthesis of plants, which is conducive to the accumulation of sugar, thereby increasing the content of reducing sugar [27].

### 3.2. Physicochemical Properties

The oil yield, AV, POV, K232, and K268 of 15 different varieties of MO are shown in Table 1. Although the average oil yield of macadamia nuts from Guangxi (29.26%), Yunnan (28.95%), and Guangdong (29.20%) were comparable, there was considerable variation among individual cultivars. Specifically, the Guangxi A4 (32.52%) and Yunnan 695 (32.33%) cultivars demonstrated exceptional performance, with significantly higher oil yields than other varieties. This indicates the importance of selecting and breeding high-yielding cultivars. AV and POV serve as critical indicators for evaluating hydrolytic rancidity in nuts and peroxide formation during lipid oxidation. These parameters are also considered essential markers for predicting the shelf-life stability of edible oils [28]. It can be seen from the table that the AV and POV ranges of MOs are 0.05–0.21 mg/g and 0.11–0.28 mmol/kg, respectively, which are significantly lower than the ISO 660:2020 [29] limits (AV ≤ 4.0 mg/g, POV ≤ 15 mmol/kg). It is worth noting that the AV and POV values of Guangdong 695 and Guangxi 695 samples are relatively high. This observation could be attributed to the unique chemical composition or physiological characteristics of 695 varieties, which makes it easier to form higher AV and POV values under specific environmental conditions, but its specific mechanism of action still needs to be further explored. Furthermore, K232 and K268, associated with conjugated dienes and conjugated trienes, respectively, are suitable parameters for the evaluation of the oxidative deterioration of the oil [30]. Table 1 shows that the K232 value of MO ranges from 0.74 to 0.83, and the K268 value is between 0.06 and 0.11. The results showed that the oxidation of MO was mainly in the primary oxidation stage, which was characterized by the accumulation of conjugated dynes, while the secondary oxidation reaction had not yet occurred obviously. This phenomenon may be attributed to the high unsaturated fatty acid content and the presence of natural antioxidant compounds in MOs [31].

### 3.3. Fatty Acid Profile of MOs

The fatty acid profile of MOs from different origins and varieties is summarized in Table 2. MOs are predominantly composed of C18:1 (56.66–62.75%) and C16:1 (12.24–18.73%), which together account for over 75% of the total fatty acids. Substantial amounts of saturated fatty acids are also present, primarily palmitic acid (C16:0, 4.77–6.69%) and stearic acid (C18:0, 2.84–6.78%), consistent with previous findings [12]. Notably, MOs contain an exceptionally high proportion of C16:1, significantly exceeding that of most other edible oils. This fatty acid has been associated with beneficial biological functions, including reduced diabetes risk and improved insulin sensitivity [32,33]. Therefore, MO can be regarded as a healthy cooking oil with potential metabolic benefits. Comparative analysis of the A4 cultivar across growing regions revealed distinct compositional differences: the Guangxi sample contained 14.36% C16:1 and 61.85% C18:1, while the Yunnan sample showed 15.76% C16:1 and 61.08% C18:1. These variations underscore the significant influence of geographical origin on the MO lipid profile, even within the same cultivar. The observed regional differences likely stem from environmental regulation of key biosynthetic genes and enzymes. For instance, the warm, rain–heat-synchronous climate with long frost-free periods in Guangxi and the low-latitude plateau climate with warm winters and cool summers in Yunnan [14] may differentially modulate the activity of fatty acid desaturases, explaining the variations in the desaturation index (e.g., C18:1/C18:0 ratio). Factors such as these climatic variations, soil nutrients, and light intensity can influence the expression of enzymes including acetyl-CoA carboxylase, fatty acid synthase, and fatty acid desaturase, thereby shaping the final fatty acid profiles [34].

Simultaneously, SFA content of macadamia nut oils from Guangxi ranged from 8.39% to 13.55%, which was generally higher than that of Yunnan samples (10.04–10.97%). For the same cultivar A4, the SFA level in Guangxi (12.68%) exceeded that in Yunnan (10.04%) by 2.64 percentage points. In contrast, MUFA contents exhibited an opposite trend, with Yunnan samples (78.76–81.83%) consistently surpassing their Guangxi counterparts (75.75–80.06%); likewise, Yunnan A4 (81.21%) showed a higher MUFA proportion than Guangxi A4 (80.06%). PUFA accounted for the smallest fraction among the three categories, ranging from 5.82% to 8.55%. Although Guangxi samples displayed slightly higher PUFA levels than most Yunnan samples on average, the exceptionally elevated values observed in Yunnan 695 (8.55%) and Yunnan 791 (7.88%) suggest that cultivar-specific genetic effects may outweigh geographical influences in determining PUFA accumulation. Collectively, UFA content in Yunnan samples (86.15–89.38%) surpassed that in Guangxi samples (84.24–87.32%). This compositional discrepancy aligns with the distinct climatic regimes of the two regions: the warm and humid conditions prevailing in Guangxi likely facilitate SFA biosynthesis, whereas the cool summer climate characteristic of the low-latitude Yunnan Plateau appears more conducive to MUFA enrichment. Ultimately, the interplay between cultivar genetic predisposition and local environmental factors synergistically shapes the distinctive fatty acid profiles of macadamia nut oils originating from different production areas.

### 3.4. Minor Constituents of MOs

As summarized in Table 3, the contents of tocopherols, squalene, and polyphenols varied considerably among MOs from different producing areas and varieties. *α*-Tocopherol was detected in all samples, ranging from 23.46 to 64.92 mg/kg, and constituted over 80% of the total tocopherols present. The highest *α*-tocopherol content was observed in Guangxi A16 (64.92 mg/kg), followed by Guangxi A4 (64.10 mg/kg). *γ*-Tocopherol was only found in Guangxi 695, Yunnan 660, and Guangdong Guire 1, while δ-tocopherol was absent in samples such as Guangxi A4, Yunnan 344, Yunnan 788, Yunnan 791, and Guangdong Guire 1. The average total tocopherol content was significantly higher in Guangxi MOs (50.44 mg/kg) than in those from Yunnan (38.49 mg/kg) and Guangdong (45.72 mg/kg). These results confirm notable regional and varietal differences in tocopherol composition, consistent with previous reports that MOs contain limited phenolic compounds [5].

Squalene content ranged widely from 66.93 to 329.01 mg/kg, substantially higher than reported in Brazilian MO (22.9 mg/kg) [35]. Regionally, Guangxi samples showed the highest average squalene content (224.60 mg/kg), followed by Yunnan (174.48 mg/kg) and Guangdong (95.89 mg/kg). Varietal differences were also evident: Yunnan 695 had the highest squalene content in its group (151.03 mg/kg), whereas Guangxi OC and A4 varieties exhibited the highest overall values (207.43 mg/kg and 329.01 mg/kg, respectively). The remarkable accumulation of squalene in Guangxi samples, particularly in A4, could be an adaptive response to environmental stressors prevalent in the region, such as high temperature and strong solar irradiance. Such conditions are reported to upregulate the mevalonate pathway and the key enzyme squalene synthase, leading to enhanced synthesis of this isoprenoid antioxidant as part of the plant’s defense system [36,37].

Polyphenol content also showed significant varietal differences. Yunnan OC had the highest level (30.27 mg/kg), followed by A4 (26.23 mg/kg), while the 695 cultivar had the lowest (10.05 mg/kg). Regionally, the average polyphenol content decreased in the following order: Guangxi (20.42 mg/kg) > Yunnan (19.82 mg/kg) > Guangdong (17.36 mg/kg). The pronounced accumulation of polyphenols in specific varieties like Yunnan OC and A4, despite the regional average being lower, highlights a strong genetic predisposition for polyphenol synthesis that can be fully expressed under conducive environmental conditions. This underscores the potential for targeted breeding of these high-performing genotypes in optimal environments to maximize the health-promoting properties of the oil.

### 3.5. Antioxidant Capacity Assays of MOs

In this study, the antioxidant capacity of MOs from different origins and varieties was systematically evaluated using OSI, DPPH, FRAP, and ABTS methods. The OSI is a key metric for assessing oil stability during storage and processing, where a higher value indicates stronger oxidative stability [38]. As shown in Table 4, Guangxi A4 MO exhibited the strongest overall antioxidant performance, with the highest OSI (18.16 h), DPPH (118.86 μmol TE/kg), FRAP (493.47 μmol TE/kg), and ABTS (825.11 μmol TE/kg) values among all samples. It also stood out in oxidation stability, far exceeding that of Yunnan 660, which had the lowest OSI (11.60 h). Regionally, MOs from Guangxi showed the highest average OSI (14.99 h), which was slightly better than those from Guangdong (14.82 h) and Yunnan (14.15 h). Notably, for shared varieties such as A4, 695, OC, and Guire No.1, Guangxi samples consistently outperformed those from other regions in OSI, further supporting the superior oxidative stability of Guangxi MOs. In broader comparisons, MOs demonstrated significantly higher OSI values than walnut oil (2.63 h) [39] and peanut oil (6.02–13.93 h) [40], reaffirming their excellent antioxidant properties. These results collectively highlight notable regional differences in MO antioxidant capacity, with Guangxi A4 showing particularly remarkable free radical scavenging ability.

### 3.6. Potentially Hazardous Contaminants of MOs

Aflatoxins, BaP, and 3-MCPDE are highly hazardous food contaminants known for their carcinogenic, organ toxic, and immunosuppressive effects, posing serious risks to human health and food safety [41,42,43,44]. Aflatoxins, as highly carcinogenic mycotoxins produced by molds, can be quantified with an LOQ of 0.5 μg/kg, as specified in ISO 16050:2003 [23]. BaP, as a representative substance of polycyclic aromatic hydrocarbons, is classified as a Group 1 carcinogen and is primarily generated during high-temperature processing of oils and fats or during grilling processes. Regulation (EC) No 333/2007 of the European Commission requires its limit of detection (LOD) to be below 0.3 μg/kg. None of the samples contained detectable levels of these contaminants, as their contents were below the detection limits. 3-MCPDE is a process contaminant formed during oil refining and heating processes, posing potential health risks. According to ISO 18363-3:2024 [45], its LOD and LOQ are set at 30 μg/kg and 100 μg/kg, respectively [46]. Although the processing method used in this study did not involve high-temperature refining, trace amounts of 3-MCPDE may still be generated during oil extraction or storage, likely due to the reaction of chlorides naturally present in the nut raw materials with triglycerides [47]. The detected 3-MCPDE content in the macadamia oils ranged from 0.07 to 4.86 μg/kg, which is substantially below the safety limit of 1250 μg/kg (Table 3), indicating that the associated risk is within a controllable range. Nevertheless, further optimization of the production process is recommended to minimize 3-MCPDE formation.

### 3.7. HCA

**Figure 1:**
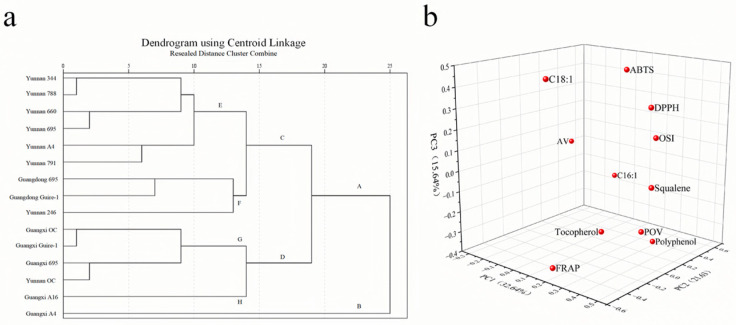
Hierarchical cluster analysis and PCA loading plot of MOs. (**a**) Hierarchical cluster analysis (HCA) dendrogram of 15 macadamia oil samples based on cosine and centroid methods. Different uppercase letters (A, B, C, etc.) on the branches indicate the sample groups identified by the analysis. (**b**) Principal component analysis (PCA) loading plot illustrating the contribution of different variables to the first three principal components (PC1, PC2, and PC3).

Clustering analysis based on cosine and centroid methods clearly grouped the MOs by geographical origin (Figure 1a). At a distance threshold of 25, the samples separated into two primary clusters: A and B. Cluster B consisted solely of the Guangxi A4 sample, distinguishing it due to its exceptionally high MUFA, polyphenol, and squalene content, along with superior antioxidant capacity (DPPH, ABTS). Further subdivision of Cluster A at a threshold of 20 yielded Cluster C (Yunnan and Guangdong samples) and Cluster D (containing all other Guangxi samples, which generally shared higher protein, tocopherol, and squalene levels). At a threshold of 15, Cluster C split into Cluster E (predominantly Yunnan varieties, often characterized by higher UFA and reducing sugar content) and Cluster F (predominantly Guangdong varieties). This clear regional clustering reflects significant differences in the physicochemical properties, nutrient composition, and fatty acid profiles of MOs.

### 3.8. Multivariate Linear Regression (MLR)

MLR was utilized to investigate the relationships between the antioxidant capacity (dependent variable Y) of MOs and their minor components (independent variables X). The general form of the linear model is expressed as Y = M_0_ + M_1_X_1_ + M_2_X_2_ + M_3_X_3_ + … + M_n_X_n_, where Y is the predicted response, M_0_ is the constant term, M_1_ to M_n_ are the partial regression coefficients, and X_1_ to X_n_ represent the content of each minor component.

As summarized in Table 5, stepwise MLR analysis identified four key components significantly correlated with antioxidant capacity: polyphenols, squalene, tocopherol, and C18:1. The regression model based on the OSI showed excellent predictive accuracy, with an adjusted R^2^ value of 0.970. Analysis of the standardized coefficients across different antioxidant models revealed distinct contribution patterns: squalene had the strongest positive effect in both the OSI (coefficient = 1.990) and FRAP (157.683) models; polyphenols contributed most significantly to the DPPH model (11.233) and showed positive effects in all models; and tocopherol exerted the most notable influence in the ABTS model (137.727). In contrast, C18:1 consistently demonstrated a negative correlation across all models, most prominently in the OSI model (−0.107). Overall, squalene, polyphenols, and tocopherol were established as the primary positive contributors to antioxidant activity, whereas C18:1 exhibited a consistent inhibitory effect.

The positive contributions of these bioactive components can be attributed to their distinct antioxidant mechanisms. Polyphenols act by donating hydrogen atoms from their phenolic hydroxyl groups to terminate free-radical chain reactions. Squalene scavenges reactive oxygen species and decomposes lipid hydroperoxides through its conjugated double-bond system. Tocopherols inhibit the propagation of lipid peroxidation by stabilizing peroxyl radicals and forming stable tocopherylquinone derivatives. Conversely, the consistent negative correlation of oleic acid (C18:1) with antioxidant metrics, despite its MUFA nature, underscores a crucial distinction: while MUFAs are more stable than PUFAs, they are not antioxidants themselves. The high abundance of C18:1 dilutes the effective concentration of natural antioxidants in the oil matrix and provides a substantial substrate pool for oxidation once the antioxidant defense is overwhelmed, thereby negatively impacting the overall oxidative stability. Based on these findings, future breeding and selection strategies for macadamia should prioritize varieties with high levels of polyphenols, tocopherols, and squalene to develop high-value industrial oils with superior oxidative stability.

### 3.9. Principal Component Analysis (PCA)

PCA was applied to assess the relationships among 11 quantitative indicators of MOs from three producing areas (Figure 1b). The first four principal components collectively accounted for 78.77% of the total variance, with individual contributions of 32.64% (PC1), 21.63% (PC2), 15.64% (PC3), and 8.87% (PC4), indicating that these components sufficiently captured the overall variability of the samples. The loading analysis showed that PC1 was predominantly associated with OSI and squalene content, while PC2 was closely related to AV, fatty acid profiles, polyphenols, and antioxidant capacity assays. The clear separation of samples from different regions in the PCA score plot, combined with these distinct loading profiles, visually reinforces the conclusion that geographical origin is a key driver of the overall compositional and stability differences in MOs. These orthogonal components collectively reflect the dual characteristics of MOs: a nutrient-dense profile rich in MUFAs and tocopherols, alongside inherent oxidation vulnerability due to the high proportion of unsaturated bonds.

The PCA-based quality ranking of the 15 MO samples revealed clear gradations (Table 6). The Guangxi A4 cultivar achieved the highest composite score, indicating superior overall quality, followed by Guangxi A16 and Guangxi OC. The leading position of Guangxi A4 was attributed to its high MUFA content (including C16:1 and C18:1), strong antioxidant capacity, and excellent safety profile. These physicochemical advantages suggest that Guangxi A4 is an ideal material for premium edible oil applications, particularly where thermal stability and health benefits are prioritized.

## 4. Conclusions

This study presents a systematic evaluation of the quality characteristics of macadamia nuts and their corresponding pressed oils across 15 major cultivars from three primary producing regions in China—Guangxi, Yunnan, and Guangdong. The results demonstrate that both cultivar and geographical origin significantly influence the physicochemical properties, nutritional composition, and antioxidant capacity of macadamia oil; however, their relative contributions differ. Geographical origin exerts a stronger influence on the overall quality profile, particularly with regard to minor bioactive components, whereas genetic background plays a more critical role in determining the fundamental fatty acid profile. This differential influence can be attributed to the regulatory effects of environmental factors—such as light, temperature, and precipitation—on the biosynthesis and accumulation of plant secondary metabolites (e.g., polyphenols and squalene), as well as to inherent differences among cultivars in the expression of genes involved in lipid biosynthesis.

The nut kernels exhibited high oil content (65.93–72.38%), and the pressed oils were rich in unsaturated fatty acids (84.24–87.95%), predominantly oleic acid and palmitoleic acid. Notably, the Guangxi A4 cultivar was identified as a superior cultivar, characterized by a high proportion of monounsaturated fatty acids (80.06%), substantial levels of polyphenols (21.79 mg/kg) and squalene (329.01 mg/kg), and exceptional antioxidant activity. The outstanding performance of this cultivar can be understood as the combined result of its genetic constitution and the favorable climatic conditions of Guangxi (warm, humid, and with ample sunlight): its genetic background likely underpins the efficient expression of enzymes involved in oleic acid biosynthesis, while the region-specific environmental conditions may have moderately induced secondary metabolic pathways, thereby promoting the accumulation of phenolic compounds and squalene. All analyzed oils exhibited a satisfactory safety profile. Multivariate statistical analysis further confirmed that geographical origin is a key determinant of oil quality. These findings provide a scientific basis for raw material selection, origin traceability, and the potential establishment of geographical indication systems, thereby contributing to industrial standardization, enhanced market competitiveness, and greater consumer confidence in Chinese macadamia oil. The identification of the Guangxi A4 cultivar as a premier raw material also paves the way for the development of high-value edible oils, cosmetic products, and nutraceuticals.

Despite these contributions, limitations include finite sample coverage, a lack of dynamic monitoring of quality changes during processing, and insufficient validation of the bioactivity of functional components. Future research should expand sampling, integrate molecular biology techniques to elucidate oil synthesis regulation, optimize processing strategies, and further substantiate the health-promoting properties of MO.

## Figures and Tables

**Table 1:** Basic information of 15 types of macadamia nut and MOs.

Cultivars	Macadamia Nut					Macadamia Oil				
	Moisture (%)	Crude Protein (%)	Crude Fat (%)	Reducing Sugar (%)	Oil Yield (%)	AV (mg/g)	POV (mmol/kg)	IV (g/100 g)	K232	K268
Guangxi A4	0.82 ± 0.27 ^c^	11.79 ± 0.20 ^a^	71.15 ± 2.54 ^a^	3.10 ± 0.35 ^b^	29.64 ± 2.30 ^abc^	0.09 ± 0.01 ^efgh^	0.26 ± 0.03 ^a^	86.45 ± 0.02 ^c^	0.80 ± 0.02 ^ab^	0.07 ± 0.00 ^b^
Guangxi A16	1.06 ± 0.09 ^abc^	12.97 ± 0.90 ^a^	72.38 ± 2.68 ^a^	2.37 ± 0.05 ^c^	32.52 ± 1.78 ^a^	0.10 ± 0.01 ^defg^	0.17 ± 0.02 ^bcd^	85.57 ± 0.01 ^fg^	0.83 ± 0.02 ^a^	0.11 ± 0.00 ^a^
Guangxi OC	1.07 ± 0.11 ^abc^	9.21 ± 0.01 ^cd^	66.70 ± 1.13 ^a^	2.62 ± 0.14 ^bc^	28.34 ± 1.57 ^abc^	0.05 ± 0.03 ^h^	0.18 ± 0.02 ^bcd^	84.78 ± 0.04 ^jk^	0.78 ± 0.02 ^ab^	0.07 ± 0.00 ^b^
Guangxi 695	1.14 ± 0.09 ^a^	9.54 ± 0.09 ^bc^	70.50 ± 3.67 ^a^	2.46 ± 0.11 ^bc^	29.69 ± 3.42 ^abc^	0.11 ± 0.01 ^de^	0.28 ± 0.00 ^a^	85.21 ± 0.03 ^hi^	0.79 ± 0.02 ^ab^	0.08 ± 0.00 ^b^
Guangxi Guire-1	1.05 ± 0.08 ^abc^	12.10 ± 0.14 ^a^	65.93 ± 3.41 ^a^	2.11 ± 0.32 ^c^	26.09 ± 1.09 ^bc^	0.09 ± 0.02 ^efgh^	0.24 ± 0.01 ^ab^	85.37 ± 0.11 ^gh^	0.78 ± 0.02 ^ab^	0.07 ± 0.00 ^b^
Yunnan A4	0.81 ± 0.05 ^c^	8.41 ± 1.26 ^cd^	71.92 ± 0.68 ^a^	2.15 ± 0.38 ^c^	29.77 ± 1.06 ^abc^	0.13 ± 0.01 ^cd^	0.16 ± 0.00 ^cd^	85.74 ± 0.04 ^ef^	0.78 ± 0.01 ^ab^	0.07 ± 0.00 ^b^
Yunnan OC	0.96 ± 0.03 ^abc^	11.55 ± 0.10 ^ab^	68.31 ± 2.73 ^a^	2.35 ± 0.45 ^c^	28.73 ± 1.37 ^abc^	0.07 ± 0.01 ^fgh^	0.23 ± 0.01 ^ab^	85.12 ± 0.00 ^hi^	0.83 ± 0.01 ^ab^	0.08 ± 0.00 ^b^
Yunnan 246	0.87 ± 0.05 ^abc^	9.62 ± 0.08 ^bc^	70.92 ± 1.23 ^a^	2.31 ± 0.38 ^c^	28.14 ± 1.25 ^abc^	0.15 ± 0.02 ^bc^	0.21 ± 0.02 ^abc^	84.75 ± 0.02 ^jk^	0.78 ± 0.03 ^a^	0.07 ± 0.00 ^b^
Yunnan 344	0.89 ± 0.16 ^abc^	9.68 ± 0.01 ^bc^	66.53 ± 5.15 ^a^	4.21 ± 0.57 ^a^	28.28 ± 2.05 ^abc^	0.10 ± 0.02 ^def^	0.13 ± 0.01 ^d^	84.72 ± 0.51 ^k^	0.80 ± 0.01 ^ab^	0.07 ± 0.00 ^b^
Yunnan 660	0.90 ± 0.18 ^abc^	8.84 ± 0.36 ^cd^	71.28 ± 3.36 ^a^	4.77 ± 0.72 ^a^	30.14 ± 1.39 ^ab^	0.09 ± 0.01 ^def^	0.16 ± 0.03 ^bc^	85.52 ± 0.01 ^fg^	0.79 ± 0.03 ^ab^	0.07 ± 0.00 ^b^
Yunnan 695	1.01 ± 0.03 ^abc^	7.18 ± 0.95 ^d^	71.09 ± 1.73 ^a^	4.47 ± 0.26 ^a^	32.33 ± 2.35 ^bc^	0.07 ± 0.01 ^gh^	0.11 ± 0.01 ^d^	86.91 ± 0.21 ^b^	0.81 ± 0.01 ^b^	0.07 ± 0.00 ^b^
Yunnan 788	0.85 ± 0.19 ^bc^	7.66 ± 1.17 ^cd^	67.21 ± 1.85 ^a^	4.86 ± 0.59 ^a^	26.78 ± 1.01 ^a^	0.15 ± 0.02 ^bc^	0.11 ± 0.01 ^d^	85.95 ± 0.03 ^d^	0.74 ± 0.02 ^ab^	0.06 ± 0.00 ^b^
Yunnan 791	1.10 ± 0.07 ^ab^	7.41 ± 1.00 ^d^	68.46 ± 2.96 ^a^	4.69 ± 0.41 ^a^	25.43 ± 1.29 ^c^	0.12 ± 0.01 ^cd^	0.11 ± 0.01 ^d^	87.46 ± 0.02 ^a^	0.78 ± 0.01 ^ab^	0.07 ± 0.00 ^b^
Guangdong 695	0.88 ± 0.15 ^abc^	4.66 ± 0.13 ^e^	70.87 ± 5.04 ^a^	4.49 ± 0.15 ^a^	30.34 ± 1.60 ^ab^	0.21 ± 0.01 ^a^	0.13 ± 0.01 ^d^	85.14 ± 0.00 ^hi^	0.77 ± 0.01 ^ab^	0.07 ± 0.00 ^b^
Guangdong Guire-1	0.84 ± 0.26 ^abc^	4.54 ± 0.25 ^e^	71.56 ± 0.61 ^a^	3.12 ± 0.34 ^b^	30.55 ± 3.18 ^ab^	0.16 ± 0.02 ^b^	0.14 ± 0.01 ^d^	84.99 ± 0.04 ^ij^	0.77 ± 0.02 ^ab^	0.06 ± 0.00 ^b^
Guangxi	1.03 ± 0.04 ^abc^	11.12 ± 0.23 ^ab^	69.33 ± 2.94 ^a^	2.53 ± 0.11 ^c^	29.26 ± 2.45 ^abc^	0.09 ± 0.01 ^def^	0.23 ± 0.01 ^ab^	85.48 ± 0.01 ^g^	0.79 ± 0.02 ^ab^	0.08 ± 0.00 ^b^
Yunnan	0.92 ± 0.03 ^abc^	8.80 ± 0.12 ^cd^	69.47 ± 3.03 ^a^	3.73 ± 0.12 ^a^	28.95 ± 1.63 ^abc^	0.11 ± 0.02 ^cd^	0.15 ± 0.02 ^cd^	85.77 ± 0.10 ^ef^	0.79 ± 0.01 ^ab^	0.07 ± 0.00 ^b^
Guangdong	0.86 ± 0.12 ^abc^	4.60 ± 0.32 ^e^	71.22 ± 3.48 ^a^	3.81 ± 0.14 ^a^	29.20 ± 3.02 ^abc^	0.19 ± 0.01 ^ab^	0.13 ± 0.01 ^d^	85.07 ± 0.02 ^i^	0.77 ± 0.03 ^ab^	0.07 ± 0.00 ^b^

All data represents the mean of three replications (Mean ± SD). The superscript letters indicate the statistical difference in rows at a significance level of 5%.

**Table 2:** Fatty acid profiles (%) of MOs.

Sample	C14:0	C16:0	C16:1	C18:0	C18:1	C18:2	C18:3	C20:1	C24:0	SFA	MUFA	PUFA	UFA
Guangxi A4	0.31 ± 0.00 ^L^	6.65 ± 0.00 ^b^	14.36 ± 0.02 ^g^	5.25 ± 0.01 ^f^	61.85 ± 0.00 ^b^	2.78 ± 0.00 ^f^	4.48 ± 0.01 ^e^	3.85 ± 0.03 ^h^	0.46 ± 0.00 ^bc^	12.68 ± 0.00 ^d^	80.06 ± 0.02 ^d^	7.26 ± 0.01 ^i^	87.32 ± 0.01 ^d^
Guangxi A16	0.32 ± 0.00 ^k^	5.69 ± 0.00 ^h^	13.75 ± 0.05 ^i^	5.68 ± 0.03 ^d^	60.21 ± 0.05 ^d^	3.47 ± 0.01 ^c^	4.48 ± 0.04 ^e^	3.74 ± 0.00 ^i^	0.48 ± 0.01 ^b^	12.17 ± 0.01 ^f^	77.70 ± 0.03 ^g^	7.95 ± 0.02 ^d^	85.65 ± 0.03 ^g^
Guangxi OC	0.40 ± 0.00 ^g^	6.39 ± 0.02 ^c^	14.02 ± 0.08 ^h^	5.16 ± 0.06 ^g^	59.72 ± 0.03 ^e^	2.79 ± 0.01 ^f^	4.57 ± 0.05 ^d^	4.14 ± 0.04 ^f^	0.52 ± 0.01 ^a^	12.46 ± 0.02 ^e^	77.87 ± 0.05 ^fg^	7.36 ± 0.03 ^h^	85.24 ± 0.04 ^h^
Guangxi 695	0.42 ± 0.00 ^e^	5.72 ± 0.00 ^g^	12.35 ± 0.02 ^L^	6.78 ± 0.01 ^a^	60.18 ± 0.01 ^d^	3.55 ± 0.01 ^b^	4.86 ± 0.00 ^c^	3.57 ± 0.00 ^j^	0.42 ± 0.00 ^de^	13.43 ± 0.00 ^b^	76.10 ± 0.01 ^i^	8.41 ± 0.01 ^c^	84.51 ± 0.01 ^i^
Guangxi Guire-1	0.38 ± 0.00 ^i^	5.94 ± 0.01 ^f^	12.61 ± 0.01 ^k^	6.24 ± 0.01 ^b^	59.19 ± 0.07 ^f^	3.26 ± 0.00 ^d^	5.23 ± 0.00 ^a^	3.94 ± 0.01 ^g^	0.53 ± 0.00 ^a^	13.09 ± 0.00 ^c^	75.75 ± 0.03 ^j^	8.49 ± 0.00 ^b^	84.24 ± 0.02 ^j^
Yunnan A4	0.36 ± 0.00 ^j^	5.74 ± 0.02 ^g^	15.76 ± 0.01 ^e^	3.56 ± 0.01 ^m^	61.08 ± 0.05 ^c^	3.27 ± 0.00 ^d^	3.47 ± 0.01 ^L^	4.37 ± 0.01 ^d^	0.38 ± 0.01 ^gh^	10.04 ± 0.01 ^k^	81.21 ± 0.02 ^b^	6.74 ± 0.01 ^j^	87.95 ± 0.01 ^bc^
Yunnan OC	0.39 ± 0.00 ^h^	5.48 ± 0.00 ^i^	17.34 ± 0.01 ^b^	4.20 ± 0.00 ^j^	60.30 ± 0.04 ^d^	1.99 ± 0.00 ^k^	3.82 ± 0.01 ^i^	4.16 ± 0.04 ^f^	0.39 ± 0.00 f^g^	10.46 ± 0.00 ^i^	81.80 ± 0.03 ^a^	5.82 ± 0.01 ^n^	87.62 ± 0.02 ^cd^
Yunnan 246	0.52 ± 0.00 ^c^	6.04 ± 0.00 ^e^	18.73 ± 0.02 ^a^	4.56 ± 0.01 ^i^	56.66 ± 0.00 ^h^	2.18 ± 0.00 ^j^	4.18 ± 0.02 ^g^	4.40 ± 0.01 ^de^	0.41 ± 0.00 ^ef^	11.52 ± 0.00 ^g^	79.79 ± 0.01 ^d^	6.36 ± 0.01 ^k^	86.15 ± 0.01 ^f^
Yunnan 344	0.71 ± 0.00 ^b^	6.04 ± 0.02 ^e^	16.74 ± 0.05 ^d^	4.21 ± 0.02 ^j^	59.13 ± 0.02 ^f^	2.44 ± 0.01 ^h^	3.84 ± 0.02 ^i^	4.63 ± 0.03 ^b^	0.37 ± 0.01 ^g^	10.95 ± 0.01 ^h^	80.50 ± 0.13 ^c^	6.28 ± 0.01 ^L^	86.78 ± 0.07 ^e^
Yunnan 660	0.81 ± 0.00 ^a^	5.25 ± 0.00 ^k^	17.02 ± 0.00 ^c^	3.95 ± 0.00 ^k^	60.30 ± 0.03 ^d^	2.39 ± 0.00 ^i^	3.73 ± 0.00 ^j^	4.52 ± 0.01 ^c^	0.38 ± 0.00 ^gh^	10.38 ± 0.00 ^i^	81.83 ± 0.02 ^a^	6.12 ± 0.00 ^m^	87.94 ± 0.01 ^bc^
Yunnan 695	0.46 ± 0.00 ^d^	5.39 ± 0.00 ^j^	12.24 ± 0.04 ^m^	4.71 ± 0.02 ^h^	62.11 ± 0.17 ^b^	4.57 ± 0.02 ^a^	3.99 ± 0.01 ^h^	4.42 ± 0.05 ^de^	0.42 ± 0.00 ^de^	10.97 ± 0.01 ^h^	78.76 ± 0.09 ^e^	8.55 ± 0.02 ^a^	87.31 ± 0.05 ^d^
Yunnan 788	0.52 ± 0.00 ^c^	5.70 ± 0.01 ^h^	15.78 ± 0.00 ^e^	3.69 ± 0.01 ^L^	61.23 ± 0.04 ^c^	3.17 ± 0.00 ^e^	3.53 ± 0.01 ^k^	4.47 ± 0.00 ^cd^	0.38 ± 0.00 ^gh^	10.28 ± 0.01 ^j^	81.48 ± 0.01 ^ab^	6.70 ± 0.01 ^j^	88.18 ± 0.01 ^b^
Yunnan 791	0.46 ± 0.00 ^d^	4.77 ± 0.00 ^L^	12.96 ± 0.00 ^j^	2.84 ± 0.00 ^n^	62.75 ± 0.06 ^a^	4.56 ± 0.01 ^a^	3.32 ± 0.00 ^m^	5.79 ± 0.07 ^a^	0.33 ± 0.02 ^i^	8.39 ± 0.00 ^L^	81.50 ± 0.04 ^ab^	7.88 ± 0.00 ^e^	89.38 ± 0.02 ^a^
Guangdong 695	0.46 ± 0.00 ^d^	6.25 ± 0.01 ^d^	15.53 ± 0.03 ^f^	5.59 ± 0.01 ^e^	58.91 ± 0.01 ^f^	3.18 ± 0.01 ^c^	4.30 ± 0.02 ^f^	3.69 ± 0.02 ^i^	0.39 ± 0.02 ^fg^	12.69 ± 0.01 ^d^	78.13 ± 0.02 ^f^	7.48 ± 0.01 ^g^	85.61 ± 0.01 ^g^
Guangdong Guire-1	0.41 ± 0.00 ^f^	6.69 ± 0.00 ^a^	15.56 ± 0.01 ^f^	6.01 ± 0.02 ^c^	57.49 ± 0.00 ^g^	2.56 ± 0.00 ^g^	5.11 ± 0.01 ^b^	3.71 ± 0.00 ^i^	0.44 ± 0.00 ^cd^	13.55 ± 0.00 ^a^	76.76 ± 0.00 ^h^	7.67 ± 0.01 ^f^	84.43 ± 0.01 ^i^
Guangxi A4	0.36 ± 0.00 ^j^	6.08 ± 0.00 ^e^	13.42 ± 0.01 ^i^	5.82 ± 0.00 ^d^	60.23 ± 0.03 ^d^	3.17 ± 0.00 ^e^	4.72 ± 0.01 ^c^	3.85 ± 0.00 ^g^	0.48 ± 0.00 ^b^	12.77 ± 0.01 ^d^	77.50 ± 0.11 ^g^	7.89 ± 0.01 ^e^	85.39 ± 0.02 ^h^
Yunnan	0.53 ± 0.00 ^c^	5.55 ± 0.01 ^h^	15.82 ± 0.02 ^e^	3.96 ± 0.00 ^k^	60.44 ± 0.05 ^d^	3.07 ± 0.01 ^e^	3.74 ± 0.00 ^j^	4.60 ± 0.00 ^b^	0.38 ± 0.00 ^gh^	10.37 ± 0.01 ^i^	80.86 ± 0.12 ^b^	6.80 ± 0.00 ^j^	87.66 ± 0.02 ^cd^
Guangdong	0.43 ± 0.00 ^e^	6.47 ± 0.01 ^c^	15.54 ± 0.02 ^f^	5.80 ± 0.00 ^d^	58.20 ± 0.07 ^f^	2.87 ± 0.00 ^f^	4.71 ± 0.01 ^c^	3.70 ± 0.00 ^i^	0.42 ± 0.00 ^ef^	13.12 ± 0.01 ^e^	77.45 ± 0.07 ^g^	7.57 ± 0.01 ^f^	85.02 ± 0.01 ^h^

All data represents the mean of three replications (Mean ± SD). The superscript letters indicate the statistical difference in rows at a significance level of 5%. SFA (C14:0 + C16:0 + C18:0 + C24:0), MUFA (C16:1 + C18:1 + C20:1), PUFA (C18:2 + C18:3), UFA (C16:1 + C18:1 + C20:1 + C18:2 + C18:3).

**Table 3:** The minor constituents (mg/kg) and hazardous substances (μg/kg) of MOs.

Sample	*α*-Tocopherol	*γ*-Tocopherol	*δ*-Tocopherol	Total Tocopherol	Polyphenol	Squalene	3-MCPDE
Guangxi A4	64.10 ± 5.34 ^a^	/	/	64.10 ± 5.34 ^b^	21.79 ± 0.19 ^cd^	329.01 ± 0.15 ^a^	0.11 ± 0.00 ^ef^
Guangxi A16	64.92 ± 7.09 ^a^	/	8.85 ± 0.65 ^c^	73.77 ± 4.36 ^a^	21.53 ± 2.17 ^cd^	185.06 ± 0.07 ^f^	0.18 ± 0.00 ^d^
Guangxi OC	28.24 ± 0.61 ^def^	/	5.11 ± 0.11 ^f^	33.35 ± 0.54 ^hi^	15.58 ± 0.19 ^ef^	207.43 ± 3.66 ^f^	0.19 ± 0.00 ^d^
Guangxi 695	23.46 ± 3.01 ^g^	5.10 ± 0.05 ^a^	10.22 ± 0.16 ^b^	38.77 ± 2.26 ^efgh^	18.90 ± 0.62 ^def^	92.31 ± 0.11 ^k^	4.86 ± 0.02 ^a^
Guangxi Guire-1	32.87 ± 1.56 ^def^	/	9.35 ± 0.25 ^c^	42.22 ± 0.85 ^defg^	24.32 ± 1.31 ^bc^	306.48 ± 0.14 ^b^	0.41 ± 0.00 ^c^
Yunnan A4	36.34 ± 1.91 ^cde^	/	9.36 ± 0.02 ^c^	45.71 ± 1.37 ^cdef^	26.23 ± 0.62 ^b^	185.37 ± 4.58 ^g^	/
Yunnan OC	38.81 ± 0.76 ^bcd^	/	7.77 ± 0.11 ^d^	46.58 ± 0.65 ^cde^	30.27 ± 2.72 ^a^	197.43 ± 0.11 ^e^	3.07 ± 0.05 ^b^
Yunnan 246	30.55 ± 3.23 ^defg^	/	7.03 ± 0.39 ^de^	37.58 ± 1.89 ^fgh^	25.87 ± 0.06 ^b^	277.01 ± 1.21 ^c^	0.16 ± 0.00 ^d^
Yunnan 344	26.51 ± 1.64 ^fg^	/	/	26.51 ± 1.64 ^ij^	20.89 ± 1.69 ^cd^	138.89 ± 0.37 ^i^	0.09 ± 0.00 ^ef^
Yunnan 660	32.59 ± 3.24 ^def^	4.60 ± 0.12 ^b^	11.29 ± 0.08 ^a^	48.48 ± 2.45 ^cd^	19.30 ± 0.60 ^de^	159.77 ± 0.48 ^g^	0.12 ± 0.00 ^e^
Yunnan 695	30.87 ± 7.91 ^defg^	/	5.56 ± 0.01 ^f^	36.43 ± 5.59 ^gh^	10.05 ± 0.41 ^g^	151.03 ± 0.05 ^h^	0.07 ± 0.00 ^e^
Yunnan 788	24.93 ± 0.46 ^fg^	/	/	24.93 ± 0.46 ^j^	10.75 ± 0.51 ^g^	66.93 ± 0.10 ^L^	/
Yunnan 791	41.68 ± 2.95 ^bc^	/	/	41.68 ± 2.95 ^defg^	15.24 ± 1.10 ^f^	219.46 ± 0.55 ^d^	/
Guangdong 695	44.99 ± 0.63 ^b^	/	6.34 ± 0.56 ^e^	51.34 ± 0.11 ^c^	18.97 ± 0.29 ^def^	110.76 ± 0.02 ^g^	/
Guangdong Guire-1	35.46 ± 1.29 ^cde^	4.64 ± 0.19 ^b^	/	40.10 ± 0.78 ^efgh^	15.74 ± 0.37 ^ef^	81.03 ± 0.02 ^i^	/
Guangxi	42.72 ± 2.45 ^bc^	5.10 ± 1.12 ^a^	8.38 ± 0.13 ^c^	50.44 ± 2.13 ^c^	20.42 ± 0.54 ^cd^	224.60 ± 1.24 ^d^	1.15 ± 0.00 ^a^
Yunnan	32.79 ± 3.25 ^def^	4.60 ± 0.28 ^b^	8.20 ± 0.15 ^c^	38.49 ± 2.03 ^fgh^	19.82 ± 0.23 ^def^	174.48 ± 3.45 ^f^	0.44 ± 0.01 ^b^
Guangdong	40.23 ± 3.02 ^bc^	4.64 ± 0.03 ^b^	6.34 ± 0.20 ^e^	45.72 ± 3.24 ^cdef^	17.36 ± 0.16 ^def^	95.89 ± 3.04 ^k^	/

All data represents the mean of three replications (Mean ± SD). The superscript letters indicate the statistical difference in rows at a significance level of 5%. “/” represents “not detected”.

**Table 4:** OSI (h) and antioxidant capacity (μmol TE/kg) of MOs.

Sample	OSI	DPPH-Nonpolar	DPPH-Oil	DPPH-Polar	FRAP	ABTS
Guangxi A4	18.16 ± 0.08 ^a^	177.61 ± 0.98 ^abc^	138.16 ± 2.46 ^a^	118.86 ± 12.17 ^a^	493.47 ± 0.30 ^de^	825.11 ± 7.51 ^a^
Guangxi A16	13.10 ± 0.08 ^def^	168.76 ± 11.27 ^bcd^	127.16 ± 4.60 ^bc^	68.03 ± 4.26 ^efg^	813.85 ± 1.31 ^a^	155.35 ± 19.53 ^f^
Guangxi OC	14.44 ± 0.01 ^cde^	159.66 ± 9.98 ^cde^	134.76 ± 0.60 ^a^	74.02 ± 1.09 ^def^	646.43 ± 3.52 ^c^	328.70 ± 9.42 ^cde^
Guangxi 695	13.62 ± 0.22 ^def^	189.90 ± 24.50 ^ab^	118.76 ± 0.92 ^e^	63.21 ± 1.02 ^fg^	564.24 ± 2.48 ^d^	295.06 ± 4.94 ^def^
Guangxi Guire-1	17.38 ± 0.24 ^ab^	146.56 ± 17.48 ^def^	127.87 ± 2.64 ^bc^	78.11 ± 1.10 ^cdef^	798.14 ± 9.12 ^ab^	419.25 ± 16.83 ^cde^
Yunnan A4	14.56 ± 0.13 ^cde^	146.55 ± 5.38 ^def^	129.87 ± 2.39 ^b^	103.34 ± 15.01 ^b^	406.07 ± 3.83 ^fgh^	339.48 ± 8.99 ^cde^
Yunnan OC	16.64 ± 0.18 ^abc^	204.64 ± 5.32 ^a^	123.61 ± 1.86 ^cd^	85.09 ± 8.54 ^cd^	490.92 ± 5.31 ^de^	353.77 ± 2.02 ^cde^
Yunnan 246	15.64 ± 0.03 ^bcd^	99.42 ± 13.87 ^h^	108.29 ± 1.23 ^f^	82.09 ± 2.77 ^cde^	411.73 ± 2.69 ^efgh^	342.65 ± 2.30 ^cde^
Yunnan 344	12.16 ± 0.41 ^ef^	127.37 ± 13.24 ^fgh^	111.09 ± 1.53 ^f^	75.60 ± 3.79 ^def^	338.51 ± 2.61 ^h^	450.48 ± 10.26 ^cd^
Yunnan 660	11.60 ± 0.07 ^f^	127.67 ± 6.52 ^fgh^	116.55 ± 1.56 ^e^	66.18 ± 4.92 ^fg^	708.71 ± 1.03 ^b^	362.33 ± 14.74 ^cde^
Yunnan 695	12.32 ± 0.34 ^ef^	136.12 ± 1.43 ^efg^	100.64 ± 0.94 ^g^	53.10 ± 5.19 ^g^	641.27 ± 1.31 ^c^	321.46 ± 2.30 ^cde^
Yunnan 788	14.42 ± 0.13 ^cde^	128.72 ± 2.77 ^fg^	110.63 ± 0.31 ^f^	78.22 ± 4.24 ^cdef^	369.37 ± 2.49 ^gh^	486.80 ± 6.20 ^c^
Yunnan 791	15.84 ± 0.06 ^abcd^	175.10 ± 2.79 ^bcd^	120.87 ± 4.88 ^de^	92.41 ± 7.48 ^bc^	449.40 ± 2.19 ^efg^	338.34 ± 3.47 ^cde^
Guangdong 695	12.26 ± 0.06 ^ef^	136.28 ± 24.88 ^efg^	125.92 ± 1.24 ^bc^	31.02 ± 4.35 ^h^	352.28 ± 0.52 ^h^	478.30 ± 1.58 ^c^
Guangdong Guire-1	15.62 ± 0.16 ^bcd^	114.13 ± 7.93 ^gh^	119.89 ± 0.62 ^de^	67.20 ± 2.18 ^fg^	380.13 ± 1.98 ^gh^	282.70 ± 16.45 ^ef^
Guangxi	14.99 ± 0.10 ^cde^	168.50 ± 3.57 ^bcd^	129.34 ± 2.35 ^h^	62.88 ± 2.01 ^fg^	663.23 ± 3.53 ^c^	404.69 ± 0.52 ^cde^
Yunnan	14.15 ± 0.12 ^cde^	143.20 ± 4.02 ^def^	115.19 ± 1.35 ^e^	79.59 ± 1.79 ^cdef^	477.00 ± 2.85 ^de^	374.41 ± 1.34 ^cde^
Guangdong	14.82 ± 0.11 ^cde^	125.20 ± 2.37 ^fg^	122.91 ± 1.02 ^de^	93.03 ± 1.58 ^bc^	366.20 ± 4.53 ^gh^	380.50 ± 1.26 ^cde^

All data represents the mean of three replications (Mean ± SD). The superscript letters indicate the statistical difference in rows at a significance level of 5%.

**Table 5:** Multiple linear regression analysis of MO and fat adjuncts.

Dependent Variable	Adjusted R^2^	Variable	R	Equation
DPPH	0.943	(Constant)	75.814	y = 75.814 + 11.233 (polyphenols) + 9.433 (tocopherol) − 0.483 (C18:1) + 0.275 (squalene)
		polyphenols	11.233	
		tocopherol	9.433	
		C18:1	−0.483	
		squalene	0.275	
ABTS	0.863	(Constant)	385.819	y = 385.819 + 0.129 (polyphenols) + 137.727 (tocopherol) − 0.160 (C18:1) + 0.468 (squalene)
		polyphenols	0.129	
		tocopherol	137.727	
		C18:1	−0.160	
		squalene	0.468	
FRAP	0.947	(Constant)	524.301	y = 524.301 + 0.138 (polyphenols) + 0.041 (tocopherol) − 0.154 (C18:1) + 157.683 (squalene)
		polyphenols	0.138	
		tocopherol	0.041	
		C18:1	−0.154	
		squalene	157.683	
OSI	0.970	(Constant)	14.517	y = 14.517 + 0.231 (polyphenols) + 0.065 (tocopherol) − 0.107 (C18:1) + 1.990 (squalene)
		polyphenols	0.231	
		tocopherol	0.065	
		C18:1	−0.107	
		squalene	1.990	

**Table 6:** Ranking of the principal component analysis scores of MOs.

Cultivar	F1	F2	F3	F4	F	Sequence
Guangxi A4	0.96	0.06	0.32	0.15	0.37	1
Guangxi A16	0.07	−0.35	−0.34	0.16	−0.12	11
Guangxi OC	0.03	−0.24	−0.02	−0.17	−0.10	10
Guangxi 695	−0.08	−0.19	−0.11	−0.02	−0.10	10
Guangxi Guire-1	0.55	−0.16	−0.13	−0.05	0.05	4
Yunnan A4	0.14	0.16	0.06	0.01	0.09	3
Yunnan OC	0.41	0.18	−0.12	−0.09	0.10	2
Yunnan 246	0.16	0.53	−0.20	−0.08	0.10	2
Yunnan 344	−0.30	0.22	0.07	−0.13	−0.04	8
Yunnan 660	−0.14	−0.12	−0.15	0.00	−0.10	10
Yunnan 695	−0.38	−0.50	0.11	−0.04	−0.20	12
Yunnan 788	−0.47	0.10	0.34	−0.03	−0.02	6
Yunnan 791	0.01	−0.18	0.29	0.03	0.04	5
Guangdong 695	−0.57	0.25	−0.07	0.25	−0.03	7
Guangdong Guire-1	−0.39	0.24	−0.04	0.01	−0.05	9

## Data Availability

The original contributions presented in this study are included in the article. Further inquiries can be directed to the corresponding author.
